# Meigs and Pepper on Diseases of Children

**Published:** 1874-04

**Authors:** 


A Practical Treatise on the Diseases of Children. *By J. Forsyth Meigs, M.D., one of the physicians of the Pennsylvania Hospital, etc., etc., and William Pepper, M.D.,* Lecturer on Clinical Medicine in the University of Pennsylvania, etc., etc. Fifth Edition, revised and enlarged. Philadelphia: Lindsay & Blakiston, 1874. Octavo; pp. 1008. Cloth, $6.00; Sheep, $7.00.

The work of Forsyth Meigs upon the Diseases of Children has been so long and so favorably known to the profession as to have established itself firmly among standard American authorities. In 1870, the author associated with himself Dr. Wm. Pepper, in a thorough overhauling and revision of the entire work and at the same time greatly enlarging it. In the fifth edition, it has been again revised and brought fully up to date. In its present form it is so complete and satisfactory that we imagine but few of our readers, who possess themselves of it, would desire any other treatise upon the important subject to which it is devoted.

